# Video Conferencing Peer Support and Rarer Forms of Dementia: An Exploration of Family Carers’ Positive Experiences

**DOI:** 10.1177/10497323231172880

**Published:** 2023-07-03

**Authors:** Jessica M. Rapley, Paul M. Camic, Emilie Brotherhood, Sebastian James Crutch, Emma Harding, on behalf of the Rare Dementia Support (RDS) Impact Study team

**Affiliations:** 1 325312 University College London, London, UK

**Keywords:** rare dementias, carers, positive aspects of caring, peer support groups, video conferencing, thematic analysis

## Abstract

Little is known regarding the nuanced experiences of family carers for people living with rare dementias (PLWRD), with no known literature exploring their positive experiences of caring discussed within peer support group settings. This article explores family carers of PLWRD’s positive experiences reported in video conferencing peer support groups. Six peer support group sessions involving a total of nine participants were qualitatively analysed using thematic analysis, guided by the conceptual framework of positive aspects of caring (CFPAC) (Carbonneau et al., 2010). Six themes were identified: (1) Protecting, maintaining, enjoying and finding strength in their relationship with the PLWRD; (2) Using tools and resources in response to challenges; (3) Positive impact of interactions and others’ responses to the dementia; (4) Overcoming barriers to taking a break while maintaining their wellbeing, (5) Maintaining positive outlooks and showing psychological resilience in adversity; and (6) Attributing meaning to the caring role. This article highlights family carers of PLWRD’s positive psychological, physical and social resources, balanced against the challenges of caring and maintaining their wellbeing, and identifies ways of promoting family carers’ positive caring experiences and resources within healthcare and supportive settings.

## Background

More than 885,000 people in the UK are estimated to be living with dementia (Wittenberg et al., 2019). Family care is the cornerstone of support for people living with dementia (PLWD) providing £8bn of unpaid care annually in the UK (Daley et al., 2019). Whilst managing their own psychological, social and physical wellbeing, family carers play an important role in supporting PLWD, managing their symptoms and adapting to life changes.

Rarer forms of dementias, characterised by non-memory-led progressive cognitive deficits, are low prevalence, more likely to be early onset and in some cases can be inherited (Table 1; Sampson et al., 2004). Caring for people living with rare dementia (PLWRD) can come with unique and complex challenges. For example, an inductive content analysis involving people affected by frontotemporal dementia (FTD) identified specific challenges with obtaining a diagnosis, families and friends trivialising family carers’ experiences, and managing complex emotional and behavioural symptoms (Bruinsma et al., 2022). A dyadic interview study of people with Posterior cortical atrophy (PCA) and their family members highlighted that the early onset, rarity and atypical symptoms of PCA led to challenging experiences, including a convoluted diagnostic journey, lack of professional awareness and dealing with uncertainty regarding how to manage ongoing changes now and in the future without specific guidance and support (Harding et al., 2018).

**Table 1.** Types of Rarer Dementias Involved in the Present Article.

Type of rarer form of dementia	Description
Familial Alzheimer’s disease (fAD)	Alzheimer’s disease symptomology Autosomal dominant penetrant genes: PSEN1 (majority), APP (rare) and PSEN2 (very rare) Commonly early onset
Behavioural variant frontotemporal dementia (bvFTD)	Typically presents with executive dysfunction, loss of inhibition or empathy, changes in appetite (particularly sweet tooth), perseverative/stereotyped/compulsive behaviours and apathy Commonly early onset
Primary progressive aphasia (PPA) with non-fluent (nfPPA), semantic (SD) and logopenic (LPA) subtypes	Language-led dementia nfPPA: Early symptoms of agrammatism, apraxia of speech and phonemic errors SD: Comprehension deficit with challenges matching words with images or meanings LPA: Impaired single-word retrieval, naming and sentence repetition Commonly early onset
Posterior cortical atrophy (PCA)	Visually led dementia with complex visual (e.g. fragmented vision) and visuospatial difficulties, spelling and calculation affected and perceptual difficulties Commonly early onset

*Note.* Information sourced from Gorno-Tempini et al. (2011), Gupta et al. (2009), Rascovsky et al. (2011) and Crutch et al. (2017).

The needs of family carers of PLWRD have been found to be often unmet by general dementia support services, which has been described as leading to frustration and lack of reassurance for the PLWRD and their family carer (Millenaar et al., 2016; Oyebode et al., 2012). Research describes a lack of professional and general awareness and understanding of rarer dementias, and also insufficient support to meet the needs associated with the specific challenges of these conditions (Bruinsma et al., 2022; McIntyre et al., 2019).

There is abundant literature focussing on the negative aspects of caring through loss-deficit and stress-process models with burden, depression and stress outcomes (Cunningham et al., 2018; Etters et al., 2008; Lloyd et al., 2016). While this perspective is important, only acknowledging this is reductionist, and consequently, the diversity of family carers’ experiences is not accounted for. It is vital to recognise the positive aspects of caring, as this will highlight potential protective factors to challenging experiences, inform support services to promote positive caring experiences and provide a new perspective to understand family carers’ wellbeing (Carbonneau et al., 2010; Peacock et al., 2009).

**Figure 1.**
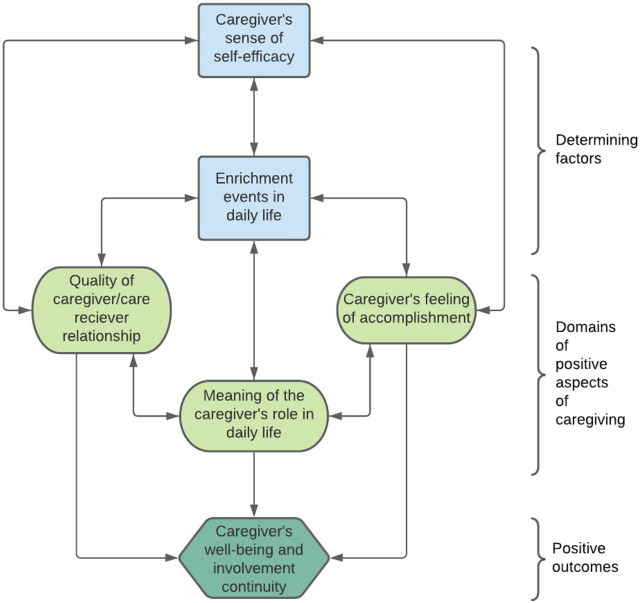
Positive aspects of caregiving model. Adapted from Carbonneau et al. (2010).

The conceptual framework of the positive aspects of caring (CFPAC) depicts factors that facilitate the occurrence of positive caring experiences, for example, carers’ sense of self-efficacy and interdependent components of positive aspects of caring, such as, quality of the caregiver/care receiver relationship, that work together to reinforce positive outcomes for carers’ wellbeing and involvement continuity (Figure 1; Carbonneau et al., 2010). The validity of this model is supported by Li and Loke’s (2013) literature review illustrating that the positive experiences of family carers of people living with cancer included an enhanced relationship with the care receiver, feelings of reward, a sense of personal growth and perceptions of personal satisfaction. Studies further demonstrate that positive aspects of caring for PLWD, such as Alzheimer’s disease, are associated with increased quality of life, life satisfaction and self-efficacy, as well as reduced depressive symptoms and burden (Daley et al., 2019).

Exploring family carers’ positive experiences provides opportunities to consider how these experiences weigh against challenges and the resulting influence on wellbeing. Dodge et al. (2012) define wellbeing as a balance between psychological, social and physical resources, drawn upon through positive experiences, and challenges. That balance reaches inevitable homeostasis at its equilibrium until disrupted by new experiences. This process may be particularly salient in dementia, with the continually changing demands of caring causing frequent imbalances and an ongoing necessity to adapt resources to meet those challenges.

Peer support groups provide an environment that promotes experience sharing, while also providing a sense of relief from caring and challenging emotions, and belonging with others undergoing similar experiences (Roche et al., 2016). This is shown to have a positive emotional and social impact, sourced from identifying with others’ experiences and feeling reciprocal support from other carers (Keyes et al., 2016). Due to the rarity, the sparse geographical distribution and the earlier onset of rare dementias, video conferencing (VC) support groups are shown to be particularly beneficial for rurally based family carers of PLWRD and those working from home in part- or full-time employment (O’Connell et al., 2014). VC peer support groups have been shown to have similar outcomes to in-person groups, providing a comfortable, safe, virtual environment to share (Banbury et al., 2019; Gentry et al., 2018), lending support that VC peer support groups may be beneficial settings to accommodate rare dementia family carers’ needs and capture their positive experiences.

As a part of a wider research study that investigates support for rarer forms of dementia (Brotherhood et al., 2020), the present study explores the positive experiences of caring reported by family carers of PLWRD in VC peer support groups. There is limited literature focussing on understanding the nuanced experiences of this population and no literature, to our knowledge, exploring family carers’ positive experiences of supporting PLWRD discussed within internet-based peer support group settings.

## Methods

### Participants and Recruitment

Ethical approval for this study was granted by the UCL Research Ethics Committee (reference no. 8545/004). All participants provided written informed consent prior to enrolment in the study. Members of Rare Dementia Support (RDS) – a UCL-led national service offering support, advice and information to people affected by rare dementia – were invited, via email, newsletters, the organisation’s website and social media channels, to take part in themed VC peer support groups. People become members of RDS via referral from their healthcare provider, signposting from charities and self-referral. While invitations to the groups were distributed to the whole membership, the sample was naturally self-selecting, and places were offered on a first-come first-served basis. A community-based voluntary sample of nine family carers (*M*_*Age*_ = 59.31, *SD*_*Age*_ = 11.27, Range_Age_ = 31.33) from six ‘Independence and Identity’–themed VC peer support group sessions was used. Of the six sessions selected for analysis, each participant attended at least two and up to four sessions. At the time of the groups starting, the PLWRD had been diagnosed on average 3.44 years previously (range .5–9 years). Carer demographics are outlined in Table 2.

**Table 2.** Participants.

Demographic information	Demographic category	N (9)
Sex	Female	7
	Male	2
Education	Further education	7
	Secondary education	1
Ethnicity	White British	5
	Irish	1
	Any other white background	1
	British Indian	1
Native language	English	8
Relationship to the PLWRD	Spouse/partner	8
	Adult child	1
Dementia diagnosis	PCA	3
	fFTD	2
	PPA	2
	FTD	1
	fAD	1

*Note.* Missing data have been excluded.

### Data Collection

Sessions were conducted remotely and recorded using an encrypted VC platform (GoToMeeting, 2021) that complied with national research security guidance. Support group sessions took place either weekly or fortnightly during mid-morning or mid-afternoon to maximise participation around family carers’ responsibilities. Each session was 1 to 2 hours in length and was co-facilitated by two staff members, using a guide based on the Mental Health America Support Group Facilitation Guide (Mental Health America, 2016). Recordings of sessions were uploaded onto the university’s data secure system and the original recording files were then deleted from the internet platform.

Sessions consisted of a combination of facilitated discussion, PowerPoint presentations, question-and-answer components and activities. The facilitators’ role involved covering the ground rules, promoting connections between group members, reflecting family carers’ feelings and responses back to the group and creating a safe space to share personal experiences. Ethical considerations regarding the groups running online included having limited access to nonverbal cues to participant mood and wellbeing, inability to check in privately aside from the group about potential distress and the potential incidental video capture of others. These considerations were addressed throughout sessions, for example, with check-ins and follow-ups. If support needs beyond the scope of the group were identified, members were referred to the organisation’s Direct Support Team for one-to-one follow-ups.

### Quality Assurance

A meeting was arranged with a former carer and spouse of a person living with PCA, to inform, sense-check and shape the study design in the early stages of the research. The aims, justification, CFPAC and methodology were presented, pre-determined open questions were asked throughout and the carer was encouraged to ask further questions. It was concluded that the area of interest chosen was perceived as a valuable and important topic and that the CFPAC was an appropriate guide for the coding framework development. This conversation provided invaluable insight into potential interpretations of the model. The results of the study were also discussed with this carer and they provided their perspective on how the themes converged with and diverged from their own experience.

Using the Morse et al. (2002) five verification strategies, the reliability and validity of the study design, data collection and analysis were considered throughout the research process (Table 3). Ongoing discussions between JR and EH ensured the application of these verification strategies.

**Table 3.** Five Verification Strategies to Ensure Rigour in the Study (Morse et al., 2002).

Verification strategy	Description	Consideration of strategies in this study
Methodological coherence	Ensure congruence between the research question and methods	**Background:** Guidance from the CFPAC **Methods:** Focus on ‘Independence and Identity’–themed peer support group data which aligns well with the research question; peer support group data allowed for unstructured discussions which allowed unanticipated insights to emerge
Appropriate sample	Participants who best represent or have knowledge of the topic	**Sampling:** Community-based sample of family carers of PLWRD; family carers of people with a broad range of rare dementias, dementia severity, care situations and geographical locations
Concurrent data collection and analysis	Iterative interaction between data and analysis	**Methods:** Familiarisation with peer support group video recordings and transcripts to inform analytic decisions; memo-writing (ongoing log of analytical thoughts and ideas); field notes (descriptions of video data that are not captured in the transcripts); reflexivity diary (to acknowledge and reflect on personal biases)
Thinking theoretically	A dynamic, cyclical process of reconfirming emerging ideas in new data	**Methods:** Familiarisation with peer support group video recordings and transcripts to inform analytic decisions; memo-writing; field notes; reflexivity diary; discussions with academics and other RDS members to reconfirm emerging ideas
Theory development	Deliberate movement between the micro-perspective of the data and the macro-conceptual understanding	**Background:** Description of CFPAC and existing literature to refer back to in the results and discussion **Methods:** Use of the CFPAC to guide coding framework within the thematic analysis **Results:** Moving between memos, transcripts, codes, themes, supporting quotes and explanatory commentary **Discussion:** Integrating results with existing literature, contextualising the CFPAC within the results, research implications and suggestions for future work

### Data Analysis

Thematic analysis was used to explore family carers of PLWRD’s positive experiences with Braun and Clarke’s (2006) methodology, used due to its flexibility and accessibility. The initial analysis was conducted by JR, an independent person to those delivering the peer support group sessions. A critical realist epistemology underpinned the research question, data collection and analysis. It was oriented towards a deductive, critical, constructionist approach; as the initial data exploration was guided by the CFPAC (Carbonneau et al., 2010), it had an interrogative nature and was based in the context of peer support group settings.

Six sessions of ‘Independence and Identity’–themed VC peer support groups were selected for analysis. Groups from this theme were chosen as they focused on the family carers’ subjective experiences and their interests, activities and sense of self, as well as those of the PLWRD. These topics were considered relevant as they align with aspects of the CFPAC and the study research question. JR ensured a good level of familiarity and working knowledge of the CFPAC and its application prior to analysis with a review of relevant literature (Carbonneau et al., 2010; Daley et al., 2019; Li & Loke, 2013). Positive experiences were broadly conceptualised as experiences that make a positive contribution to family carers’ daily living. Field notes were written during familiarisation with the video data and transcripts to take note of nodding, significant changes in facial expression and other notable gestures or nonverbal behaviours (e.g. clapping). The use of video data permitted rich transcription in which nonverbal behaviours such as laughter could be captured. The process of coding the six transcripts was supported by NVivo12 (QSR International Pty Ltd, 2018) and the coding framework was guided by the CFPAC (Carbonneau et al., 2010). Themes were generated and reviewed based on their relevance and coherence, and to ensure that there was not too much overlap between them, that is, that while they were related and connected, they were sufficiently and identifiably distinct from one another. Codes were reallocated and reorganised where appropriate by JR and discussed with EH and PC. The cyclical process of reviewing the initial data, memos and the research question resulted in further pruning and reorganisation of the coding framework until the final themes and codes were defined and named. A thematic map was developed to support the process of reviewing the themes to ensure their conceptual distinctiveness, despite points of linkage. Throughout, memos, field notes and a reflexivity diary were used to monitor analytic ideas and researcher assumptions.

## Results

**Figure 2.**
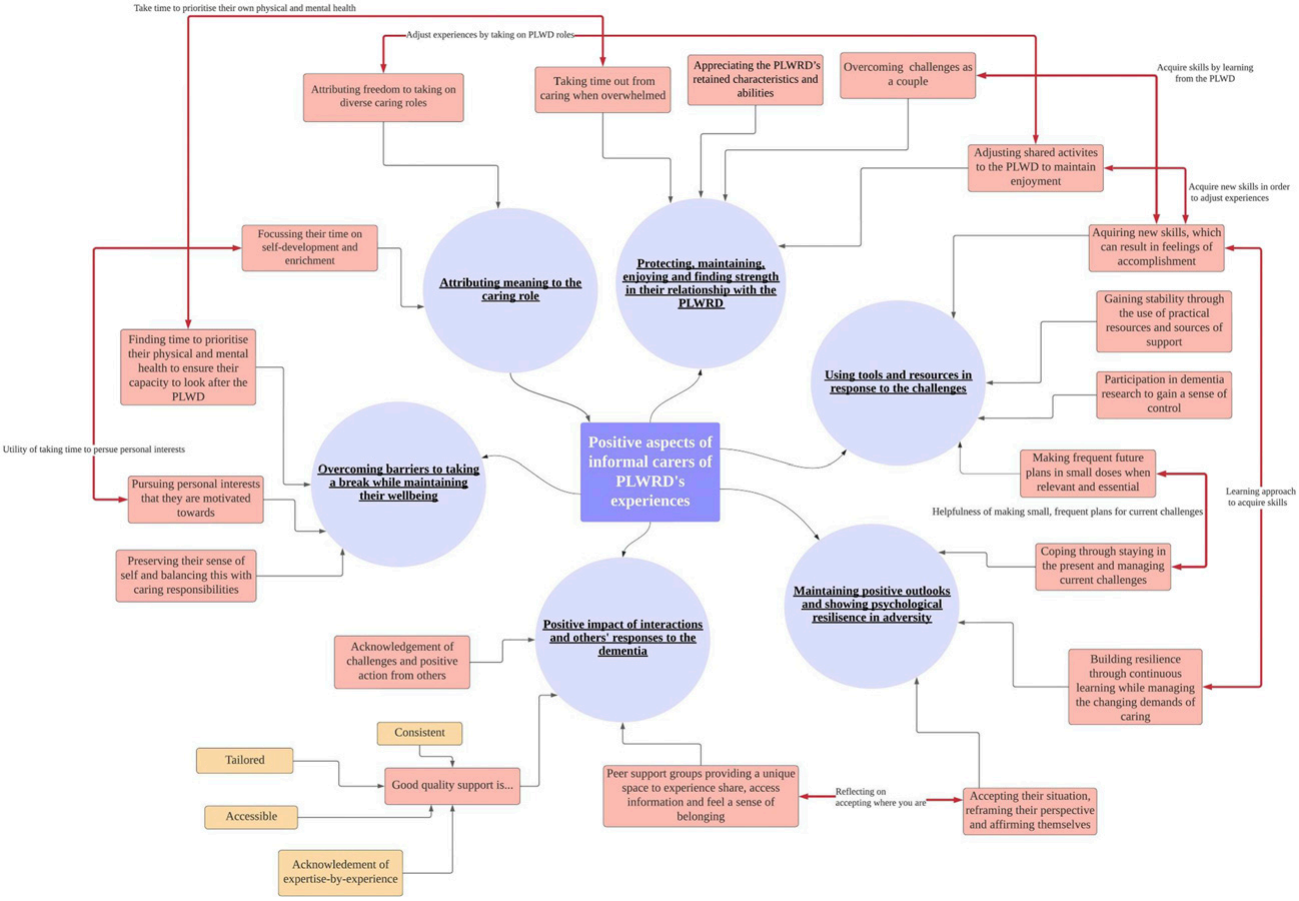
Overview of the positive aspects of informal carers of PLWRD’s experiences – themes, subthemes and links between them.

Six themes were generated from the thematic analysis: (1) Protecting, maintaining, enjoying and finding strength in their relationship with the PLWRD; (2) Using tools and resources in response to challenges; (3) Positive impact of interactions and others’ responses to the dementia; (4) Overcoming barriers to taking a break while maintaining their wellbeing; (5) Maintaining positive outlooks and showing psychological resilience in adversity; and (6) Attributing meaning to the caring role. A detailed description of each theme and subtheme along with some illustrative examples of the points of connection between them are provided below. An overview of the themes, subthemes and connections can be found in the thematic map in Figure 2.

### Protecting, Maintaining, Enjoying and Finding Strength in Their Relationship with the PLWRD

This first theme reflects how family carers nurture, adapt to, and retain their relationship with the PLWRD. This is described by family carers as helping kindle positive, enjoyable moments and experiences between the family carer and PLWRD.

Family carers reported that they protect their relationship by focussing on and appreciating the PLWRD’s retained characteristics and abilities: ‘Yes, he hasn’t lost all his independence yet but a large portion of it but he still has his sense of humour… so I treasure that’ (P9).

Family carers discussed that being able to take some time out of caring (e.g. when feeling overwhelmed) was an important factor in maintaining their relationship with the PLWRD as it allowed them to be in a better headspace when they were spending time with their relatives: ‘When you see them, because you haven’t had all that exhaustion and all that day-to-day craziness, what you normally have, you can go in it much more patient, relaxed and chilled out’ (P1).

Family carers discussed shared activities and roles taken in those activities being re-adjusted depending on the ability of the PLWRD in order to make the activities still enjoyable and possible for both of them to take part in (e.g. by taking on the PLWRD’s typical responsibilities): ‘This is one of the things we can still do together, is travelling around, at present. Just our roles have changed and I’m the driver now, (Laughter) but anyway, at least we can still do it. … It gives us freedom. … It is something we both really enjoy still’ (P9).

Spousal carers described a sense of longstanding togetherness and connectedness as a key part of their approach to overcoming challenges and continue moving forwards beyond challenges: ‘So, we’ve had those things to deal with, but as a couple we’re still here, carrying on. I think we’re overcoming those difficulties’ (P7).

### Using Tools and Resources in Response to Challenges

This second theme reflects practical, physical and psychological tools and resources used by family carers. They discussed different tools and resources used to manage day-to-day activities, the PLWRD’s symptoms, their own wellbeing and coping with the future.

Family carers communicated the necessity to acquire new skills to manage daily living, which they described as resulting in feelings of accomplishment: ‘If you don’t try these things, you sit and look at them every day too, and they drive you nuts. Or you have to go and get someone to do something so trivial that they don’t always want to come out and do it…You feel pleased with yourself if you’ve accomplished what you set out to do, so it’s not all negative’ (P9). This links to the previous theme where family carers described needing to acquire new skills to take on the PLWRD’s roles and the value of completing tasks together.

Family carers recounted using a range of practical resources and sources of support, for example, medication, mobility aids and online forums: ‘He got meds that helped and now he’s very good…he’s in more in a stable kind of place and for me, I’m back to myself’ (P8). These resources helped to provide a sense of stability in times of challenge, including helping to manage the PLWRD’s symptoms, aiding the mobility of the PLWRD within the community and supporting carer wellbeing through emotional release on online forums.

In addition, family carers reported that it was important to find the right balance with how they accessed tools and resources (e.g. information informing future planning): ‘You might read up a bit about it and take in a bit. It gets you down. You take what you need from that bit. Sometimes that helps you get a bit stronger and other times it doesn’t’ (P9). This highlights that it can be helpful for carers to make small, frequent future plans by taking on relevant, essential information and discarding overwhelming information.

One adult child carer living at risk of an inherited dementia described how actively seeking participation in dementia research provided them with a sense of control over the condition: ‘The only way you can take control… the only way I feel like I can do something about all of this and feel less inept, and a little bit more pragmatic, is to get involved in the research’ (P6). This was described as a helpful way to manage associated negative emotions and use practical resources available in response to the challenging situation.

### Positive Impact of Interactions and Others’ Responses to the Dementia

This third theme reflects the elements of good quality support for family carers of PLWRD and the impact that others’ responses can have on family carers’ everyday life experiences. This theme also captures the beneficial experiences family carers gain from interacting with other family carers in protected spaces, for example, peer support groups.

Good quality support is described by family carers across four subthemes:

***Tailored –*** Family carers reported the importance of support that is tailored to the PLWRD. Specialised support was perceived as much more beneficial than generalised support, as it addresses specific needs: ‘If they use the specialist teams, it can be much more beneficial than just saying, “We will send a carer round,”… it would be good for the sensory team to come round’ (P2).

***Consistent –*** Family carers also commented on the importance of support that is consistent, to prevent them having to continually readjust to changes in support availability, and the added challenge of finding this consistency alongside the relevant knowledge and awareness of rare dementias: ‘Our GP’s quite good but he only works two days a week… it’s really hard to get someone else and they don’t know what PCA is’ (P9).

***Accessible –*** Family carers described the importance of support that is easily accessible in times of need and on a regular basis: ‘My occupational health contact has called us regular as clockwork…They are genuinely on the end of a phone if you want to call them at any time’ (P3).

***Acknowledgement of expertise-by-experience*** – Carers attested to the importance of support which capitalises on the knowledge and expertise that family carers have of the PLWRD, so that their needs can be most adequately met: ‘Carers are best placed to deal with their loved ones. They make their loved ones feel safe. They know their intricacies’ (P1).

Others’ responses (e.g. professionals, those close to them and strangers) were described by family carers as having a positive impact due to others proactively acknowledging family carers’ challenges and responding sensitively, openly and with helpful action: ‘Connecting with people that would say, “Bless you” or comfort you with an expression, or with a hand, just a gesture… I do find it nice when people acknowledge what you’re going through’ (P8).

The ‘Independence and Identity’–themed VC peer support group sessions were described by family carers as beneficial as they provided a sense of belonging, as well as a space to access information, share experiences and express vulnerabilities without social boundaries: ‘I just think the connection we have, because when I’m with my friends, I suppose, for the most part, you put on a brave face…but this is the time where, when we connect like this, that I suppose it’s very accepting of where we are’ (P8).

In the sessions, many family carers acknowledged each other’s experiences by nodding and expressing compassion and empathy towards each other’s situations, noting that they had ‘the same problem’ (P4) and that they could ‘relate to that so much’ (P8).

Listening to others in similar situations seemed to affirm their feelings and reduce feelings of isolation: ‘You’re not the only person who’s having the same thoughts and feelings and to hear different perspectives. You learn things from each other, as well’ (P3). Sessions appeared to provide opportunities for family carers to learn, and gain ideas and resources from each other.

### Overcoming Barriers to Taking a Break while Maintaining Their Wellbeing

This fourth theme reflects how family carers have overcome barriers to balancing the PLWRD’s care needs, family carers’ personal interests and goals, and their own emotional and physical wellbeing. Family carers discuss this being a challenging balance and how in some situations it is important to prioritise some aspects of life over others.

Family carers described the need for balance between their caring responsibilities and preserving their identity and sense of self through work or other purposeful activities. However, sometimes this balance was reported as unattainable: ‘I stopped working in December, because I needed some time to take stock really, but I am definitely feeling that I need something else purposeful to do to keep a balance and have a feeling of independence and that I exist for more than one purpose’ (P3).

Family carers discussed pursuing personal interests to switch off from the caring role, restoring wellness and creating personal goals, which were described to help with optimistic thinking about the future: ‘I miss studying…So, I want to do that… Just something which I think might just refresh me and recharge me a little bit and give me something to hope for, something to personally aim for’ (P1). It was also described as helpful for family carers to gain skills and knowledge of personal interests to be pursued in the future when it will become more of a necessity to help cope with grief and loss: ‘If I do a competent sailing course, it doesn’t mean I am going out every weekend from now on to go sailing, but it is there in my back pocket. So, that when there is a void, I have got something that will help pick up the pieces and get on with things’ (P2).

Family carers mentioned that their responsibilities could deplete physical and cognitive resources, resulting in them needing to spend more time looking after their own health and less time pursuing personal interests or goals so that they could continue to support the PLWRD: ‘It is just having to think through the day and do everything through the day and plan ahead that is the most tiring… It is going to catch up with me, and that is where I think it is important to look after myself first in order to look after my wife’ (P2). This interlinks with the first theme, where family carers discussed the importance of prioritising their health to ensure that they are able to care for the PLWRD.

### Maintaining Positive Outlooks and Showing Psychological Resilience in Adversity

This fifth theme reflects family carers’ abilities to hold a positive mindset despite challenges, to build their resilience through learning during changing demands and to manage current challenges by staying in the present.

Family carers appeared to show positive outlooks by accepting the situation, reframing their perspective and affirming that they were doing the best they could, given the situation and resources available: ‘I came to a place where I accepted this was our diagnosis and I found there was actually freedom in it because all the time at the beginning I was fighting it and how unfair it all was… When I came to acceptance of it, I saw really so much more positives’ (P8). This links to the third theme that described peer support groups as settings for family carers to reflect on acceptance of the situation and to affirm one another.

Family carers reported building resilience through having to continue learning while managing the changing demands of caring: ‘We just had to learn on the go. Then, by the time we knew how to do it, she had changed again anyway’ (P6). This connects to the second theme, as family carers discussed taking a learning approach to help acquire the skills to manage daily living.

Many family carers described finding it useful to mentally stay in the present by taking ‘each day as it comes’ (P1) and ‘not staying too much ahead’ (P9). Family carers discussed that it was useful to focus on managing current challenges rather than projecting to unknown challenges ahead: ‘I try as much as possible to keep it in the day… I think the worst part about it is to project forward’ (P2). This links with the second theme where family carers describe the helpfulness of accessing relevant tools and resources to manage current challenges as they come.

### Attributing Meaning to the Caring Role

This sixth theme reflects family carers making positive attributions to the caring role. This is described by family carers to support finding constructive meaning in caring.

Family carers appeared to attribute meaning to the role by describing how they found ways to focus on their self-development and enrichment: ‘Even though I don’t see my identity as in caring, I have found it very enriching. Because I have done a lot of self-development. I put my time into enriching my life in other ways’ (P8). This interlinks to the fourth theme which describes that focussing on personal interests can enrich carers’ lives and encourage self-development, in the same way that connecting to the caring role can.

Family carers also seemed to attribute freedom to taking on diverse caring roles: ‘Yes, there are lots of roles you pick up along the way. I could go on and on about them but if you don’t do them, you don’t go out and do things, so you’ve got to do them’ (P9). This ties in with the first theme, as family carers described how taking on new caring roles, for example, the PLWRD’s role, can support positive experiences, that is, enjoying shared activities.

## Discussion

Exploring the positive experiences of family carers of PLWRD reported in ‘Independence and Identity’–themed VC peer support groups using thematic analysis generated six themes. Family carers’ positive experiences included family carers finding positives in their relationship with the PLWRD; using tools and resources; experiencing compassionate interactions and support; balancing caring responsibilities with personal life and wellbeing; maintaining positive outlooks and resilience; and attributing positive meaning to the role.

The themes generated offer new insights into the ways the determining factors and domains of the CFPAC are experienced by family carers of PLWRD (Carbonneau et al., 2010). Family carers showed self-efficacy through their positive outlooks, psychological resilience and using a learning approach to caring. Enrichment events were illustrated by family carers finding stability through utilising resources in challenging situations, enjoying shared experiences with the PLWRD and taking time to preserve their sense of self and pursue personal interests. This is described to benefit family carers by restoring wellness, allowing for personal growth and gaining skills (Peacock et al., 2009). The quality of their relationship with the PLWRD was maintained by family carers focussing on the PLWRD’s retained characteristics and abilities, adjusting enjoyed shared experiences, and psychologically and physically working together to overcome challenges. Adapting to and being flexible around PLWRD’s needs is shown to be important to support coping and planning (Kindell et al., 2014). Acquiring skills to manage the changing demands of caring was discussed by family carers to be challenging, but learning new skills and taking on new roles helped to promote resilience and provide a sense of accomplishment. Family carers could attribute positive meaning to the role by focussing on development and enrichment and finding freedom in their diverse caring roles.

Family carers also highlighted positive caring experiences that are not considered in the CFPAC. The availability of external support that is tailored to the PLWRD, accessible in times of need, consistent, and provided by people with sensitive, open and helpful attitudes, was seen as valuable to family carers. Studies highlight that tailored support is perceived to be more beneficial than generalised support, which converges with the opinions highlighted in this study (Harding et al., 2018; Millenaar et al., 2016). Family carers also described the benefits of focussing on the present and current challenges by using relevant and essential information for short-term future planning. This finding is in line with research illustrating that family carers of PLWRD viewed timely information, access to appropriate advice and consistent and flexible services as important (McIntyre et al., 2019; Sutcliffe et al., 2015), but also highlights the complexity of providing this when needs and experiences relating to dementia symptoms and age of onset can vary, as seen in the current sample.

This study has also shed light on some of the unique impacts of rare dementias due to their mostly young age of onset, atypical symptomology and potential heritability. The impacts of an earlier age of onset were illustrated by family carers’ experiences of challenges with balancing their caring role with their employment. Although employment could be lost, some family carers found balance by seeking a purposeful activity to maintain their independence. The specific impacts of the atypical symptomology were evidenced in the ways family carers were continually learning and adjusting to an unknown future. Owing to a lack of awareness of the unusual symptoms and how they would progress, carers found themselves becoming experts, with standard dementia care planning guidance inappropriate for their family member’s specific needs. Carers valued and expressed a need for specialised support, thus highlighting, as in previous studies, the specific impacts of the atypical symptoms associated with rarer dementias (Bruinsma et al., 2022; Harding et al., 2018). Others being aware of and having sensitive, open and helpful attitudes towards rare dementias, and clinicians having a comprehensive understanding of atypical symptoms were also discussed to be particularly important. The unique challenges and emotional impact that comes with genetically inherited rare dementias were highlighted by an adult child of a PLWRD, who discussed attempting to gain a sense of control over the condition’s potential future impact on her through actively being involved in dementia research, while simultaneously caring for an affected parent.

This study further contextualises positive experiences as having to be protected from, maintained alongside and balanced with the challenges, burden and stress of caring and maintaining family carers’ own wellbeing. Family carers described that there can be points where they must prioritise their own wellbeing over their caring responsibilities and personal pursuits in order to continue being able to care. This highly resonates with the Dodge et al. (2012) definition of wellbeing. The themes encompass the psychological (positive outlooks and resilience; positive attributions to the caring role), physical (using tools and resources; overcoming barriers to taking a break) and social (quality of their relationship with PLWRD; quality of interactions with others) resources family carers use and how constantly changing situations can tip the balance, requiring new resources to manage new challenges.

The VC ‘Independence and Identity’ RDS peer support group sessions were described as positive experiences in themselves, as they provided a unique online space for family carers to access information, feel a sense of belonging, and share experiences, vulnerabilities, and challenges. This aligns with previous literature that illustrates that peer support groups for family carers of PLWD help to reduce feelings of isolation (Greenwood et al., 2017), provide a chance to identify with other family carers and their experiences (Keyes et al., 2016), and offer opportunities for feelings of shared experience, a relaxing, safe environment and a space to be vulnerable without having to save face (Greenwood et al., 2013; Roche et al., 2016). VC peer support provided an opportunity for family carers to share with others with similar experiences and to gain ideas and resources, which is of particular value for those affected by rarer conditions that may not have access to people experiencing similar situations in close physical proximity (O’Connell et al., 2014; Roche et al., 2016).

### Limitations

Using VC support group data did not allow for focussed questioning or follow-up questions to probe for more details on a topic of interest. Therefore, there may be positive aspects of caring that were not captured within this thematic analysis. However, using naturalistic peer support group source data did identify shared experiences of family carers of people with a variety of rare dementias and offered a level of authenticity in members’ contributions. Although collecting, storing and analysing video-recorded data was resource-heavy, it permitted deeper, more holistic analysis of nonverbal behaviours and the social contexts captured within the field notes and also aided researchers’ interpretation in instances where textual data was ambiguous, for example, detecting sarcasm and responses directed to specific members of the group.

The sample was a small, self-selecting one, primarily of spousal family carers, and provides only limited information about adult child family carers. Thus, the variety and depth of the positive experiences of being adult children of PLWRD may not be well captured in this thematic analysis. Additionally, the VC element of the group may have led to some people not taking part due to technological barriers.

Using a deductive approach focused on the positive aspects of experiences potentially limited our understanding of the complexities of how these positive experiences interact with more negative aspects of the caring experience within contexts of different caring stages and changing family circumstances. Having said that, the fact that the themes – despite being positive in their focus – incorporated aspects such as challenges to implementing self-care and a context of adversity which demanded resilience, highlights the nuanced intersection of these positive and negative aspects of experiences as a worthwhile area of consideration for future work.

## Future Research and Applications

Future research could utilise different qualitative data sources and methods to advance the current understanding of rare dementia family carers’ experiences and subsequent outcomes. To explore the positive aspects of caring in more depth, interviews or focus groups with structured or semi-structured questioning could be used across larger samples of family carers supporting people with different forms of rarer dementias. Additionally, the positive experiences of family carers with parent–child relationships could be specifically explored to gain a richer understanding of their experiences. Future research could also explore how time spent living with the PLWRD from diagnosis influences caring experiences, although it is worth noting that PLWRD often face a convoluted journey to diagnosis and many carers reported symptoms starting years before a formal diagnosis was made.

Supporting previous literature, this study highlights a necessity for clinicians, family, friends and the general public to gain awareness of rare dementias and sensitive, open and helpful attitudes towards those affected by them. Family carers have suggested that support which can be flexibly accessed (e.g. specific rare dementia information accessible online) and in general supportive interactions with health and social care professionals, which are characterised by compassion, listening, a willingness to learn more about rare dementias and acknowledgement of the challenges family carers are facing, would all be beneficial.

The findings suggest there are benefits in health and social care professionals providing relevant, timely and essential information tailored to current challenges, which aligns with research findings in rare dementia and other chronic rare conditions (Jaffe et al., 2009; McIntyre et al., 2019). This also links in with family carers’ discussions that it is helpful to make small, frequent plans and suggests the importance of professionals providing essential, relevant information to support family carers make specific plans for the near future without overwhelming them. This could be implemented in clinical settings to better support people affected by rare dementia and has the potential to be applied more broadly to support people affected by a range of rare conditions.

This study captured how family carers demonstrate their psychological resilience, adaptability, propensity to find positives in challenges, resourcefulness in identifying ways to nurture their physical and emotional wellbeing, and creativity to utilise tools and resources beneficially. Family carers also highlighted the utility of their expertise on how to care for their family members. These findings can be used to inform the development of intervention content and resources to support and promote family carers’ wellbeing. Peer support services could draw upon family carers’ knowledge and experiences, positive mindsets and adaptability, which could be a particularly powerful way to connect family carers coming from similarly unique situations and bring light to new approaches and ways of thinking. This may be particularly helpful for those supporting PLWRD and facing the specific challenges that come with lesser-known diagnoses.

## Conclusions

This study highlights rare dementia family carers’ positive experiences discussed in VC peer support groups. Psychological, physical and social resources were highlighted in VC peer support group discussions along with the ways these resources provided balance against challenges and burden. Further research could continue to explore this topic in more depth by utilising different qualitative data sources and methods. Moving forwards, raising awareness of rare dementias, providing tailored support for family carers and harnessing family carers’ strengths in supportive and clinical settings are important steps towards promoting family carers’ positive experiences.
